# Supplementary material for: Genome-wide meta-analysis identifies novel risk loci for uterine fibroids within and across multiple ancestry groups
Source: Nat Commun. 2025 Mar 6;16:2273. doi: 10.1038/s41467-025-57483-5 (PMC11885530; doi:10.1038/s41467-025-57483-5)
Supplement: Supplementary file 1 — Supplementary Information [file 41467_2025_57483_MOESM1_ESM.pdf]

Genome-wide meta-analysis identifies novel risk loci for uterine fibroids within and across multiple ancestry groups

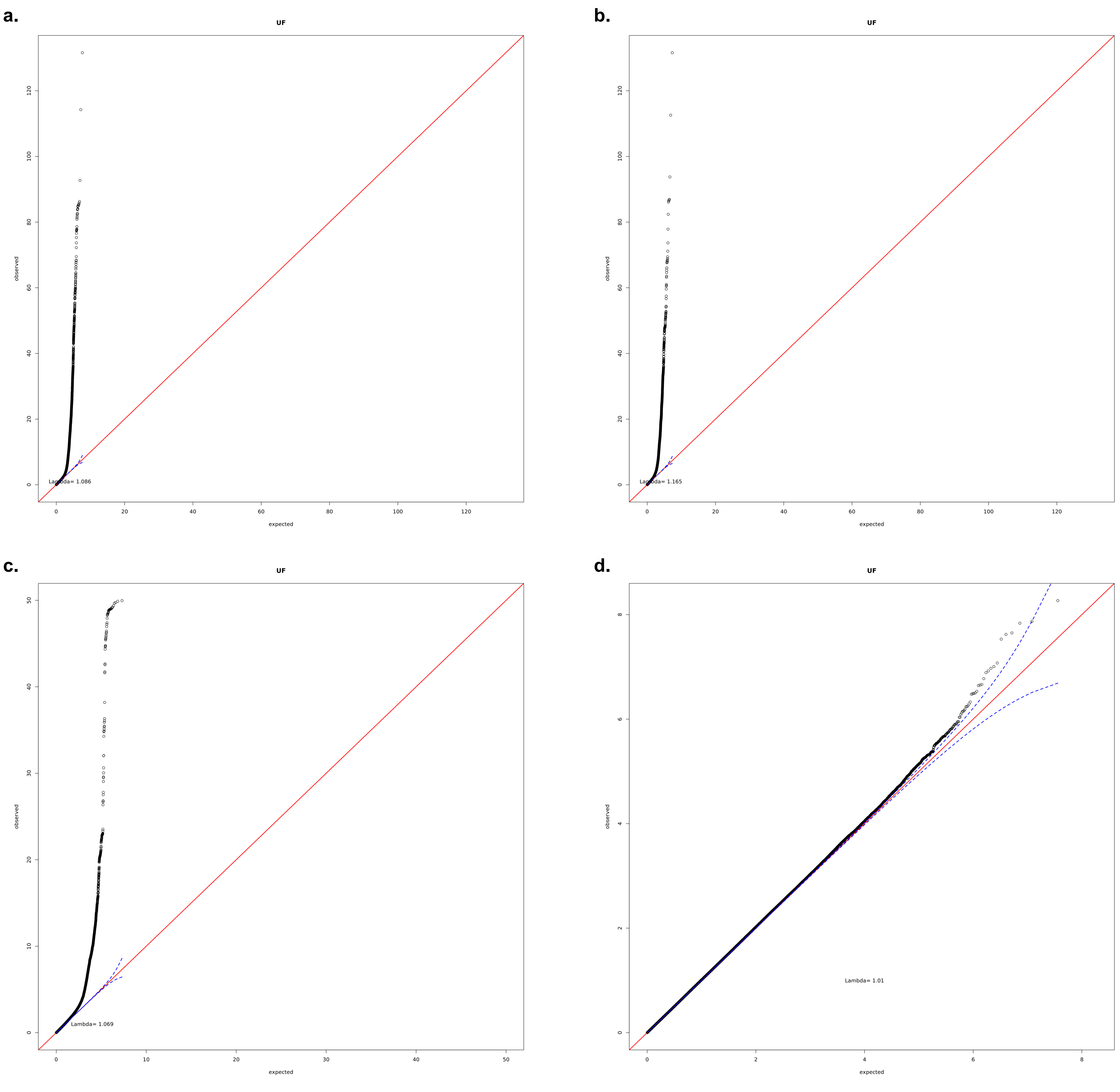

Supplementary Figure 1. Quantile-quantile (QQ) plots of meta-analyses with lambda values. a = Multi-ancestry, b =European ancestry, c = East Asian/Central South Asian ancestry, d = African ancestry. Lambda value calculated from median chi-squared values.

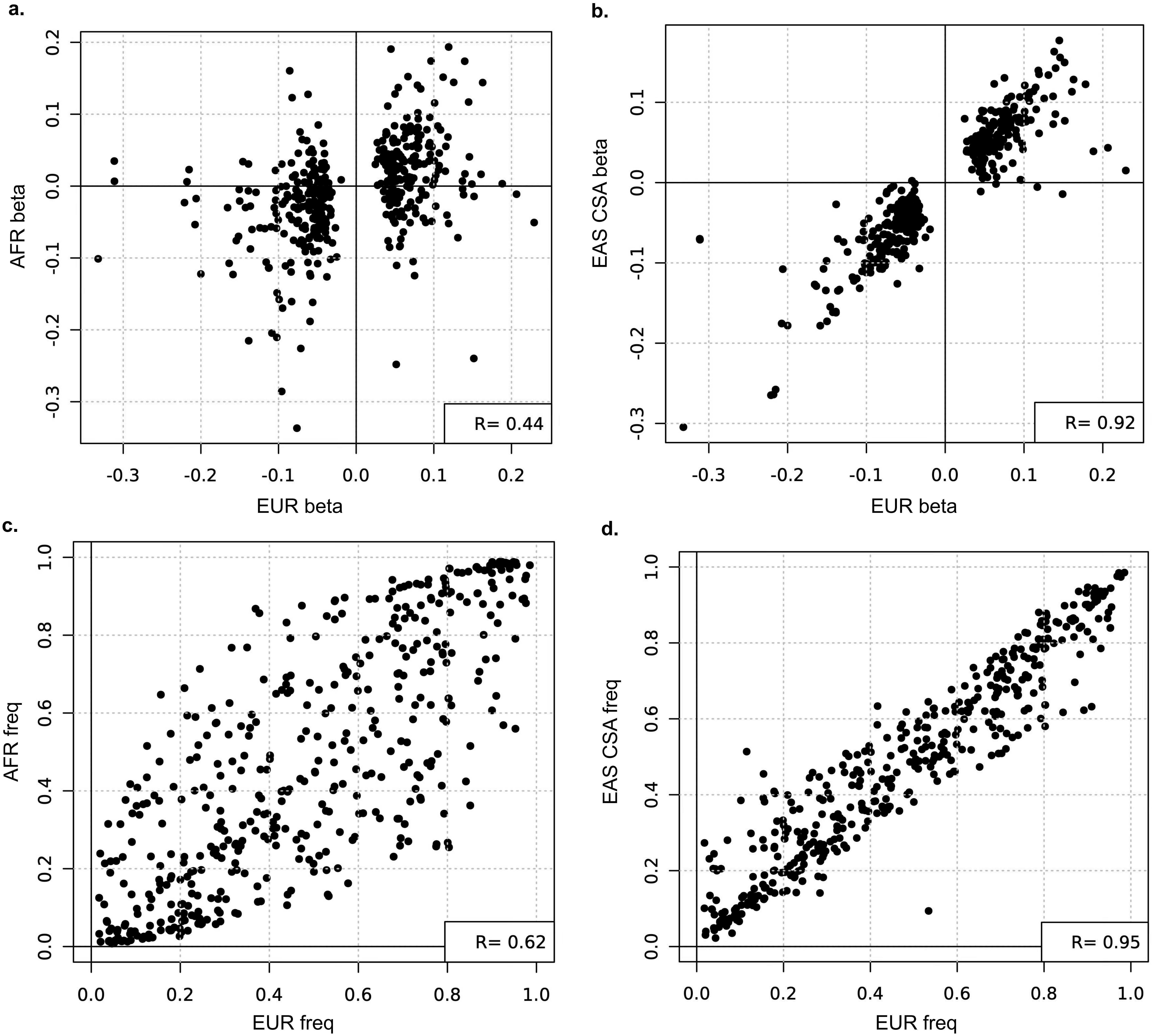

Supplementary Figure 2. Effect and frequency plots between ancestry groups for variants limited to those that were in the multi-ancestry summary statistics. a = effect size comparisons between African ancestry and European ancestry, b = effect size comparisons between East Asian/Central South Asian ancestry and European ancestry, c = effect allele frequency comparisons between African ancestry and European ancestry, d = effect allele frequency comparisons between East Asian/Central South Asian ancestry and European ancestry. Correlation statistical test with R function corr.

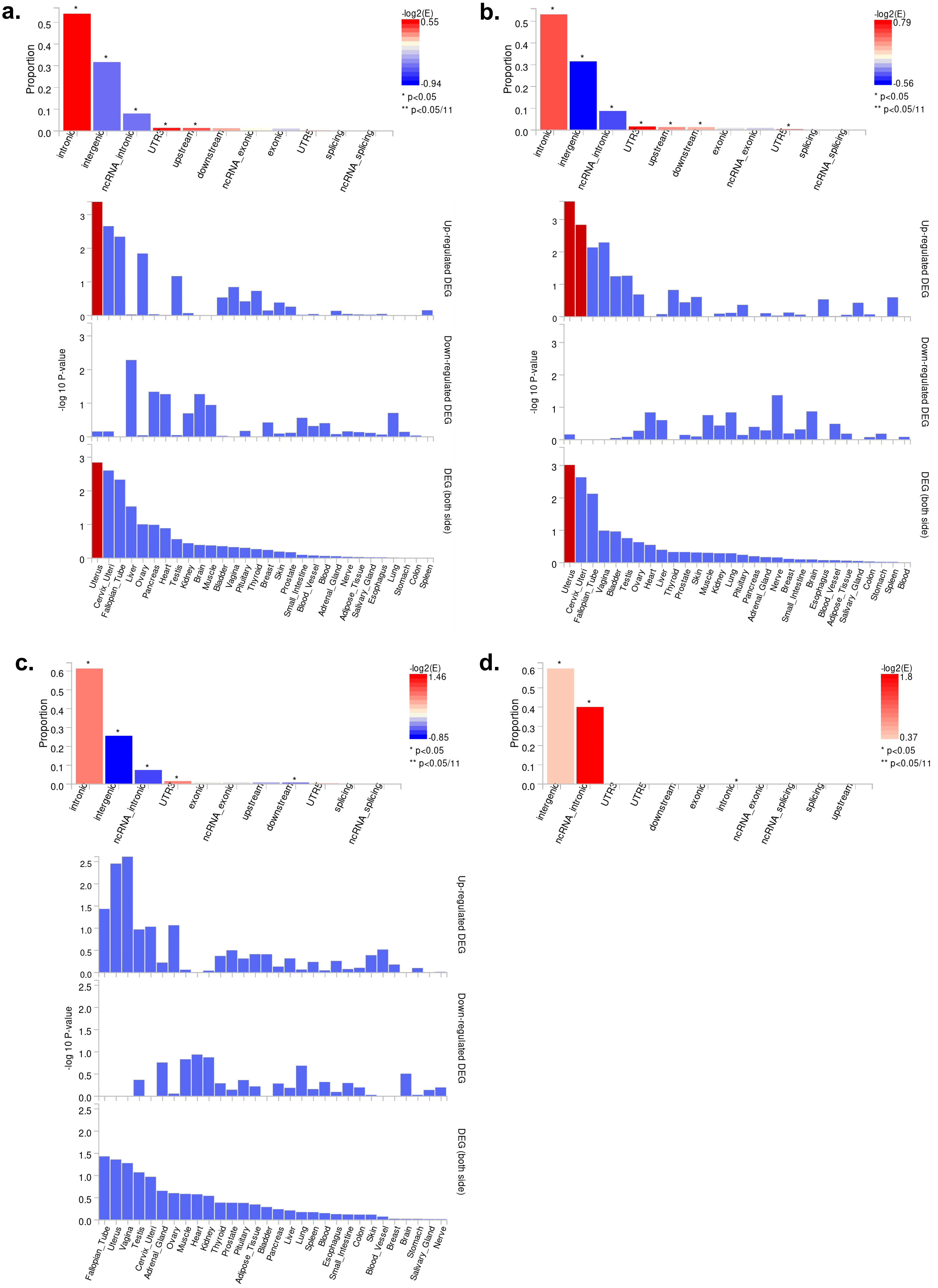

Supplementary Figure 3. Functional Mapping and Annotation (FUMA) results with annotated significant variants results of enrichment (upper panels) and differentially expressed gene enrichment (lower panels) tests. a = Multi-ancestry, b = European ancestry, c = East Asian/Central South Asian ancestry, d =African ancestry. Enrichment tests, empiric p-value threshold (0.05).

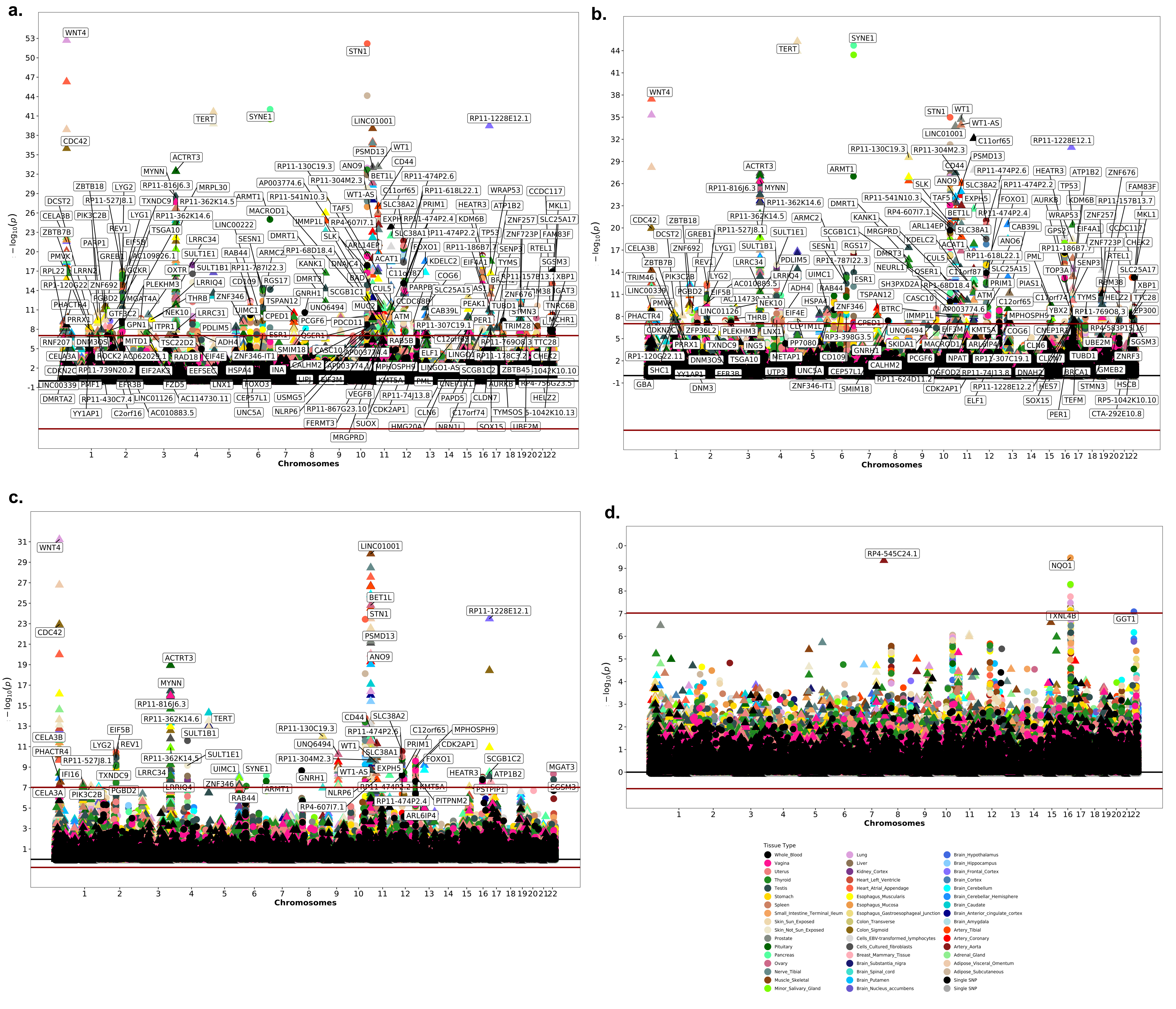

Supplementary Figure 4. S-PrediXcan results for 49 GTEx v8 tissues using JTI models. a = Multi-ancestry, b =European ancestry, c = East Asian/Central South Asian ancestry, d = African ancestry. Two-sided Wald test; multiple testing correction p-value threshold used (9.4×10^{-8}).

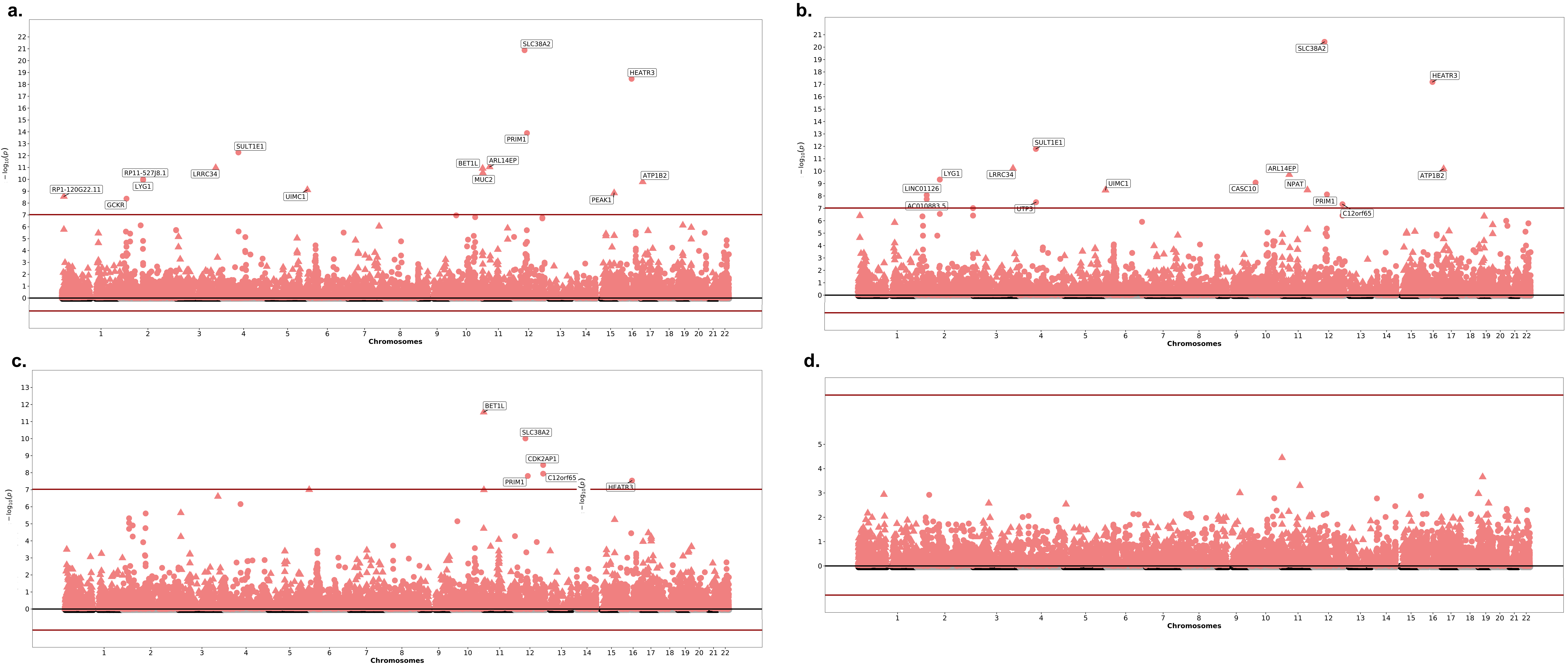

Supplementary Figure 5. S-PrediXcan results for the uterine tissue shown using JTI models. a = Multi-ancestry, b = European ancestry, c = East Asian/Central South Asian ancestry, d = African ancestry. Two-sided Wald test; multiple testing correction p-value threshold used ($9.4x10^{-8}$).

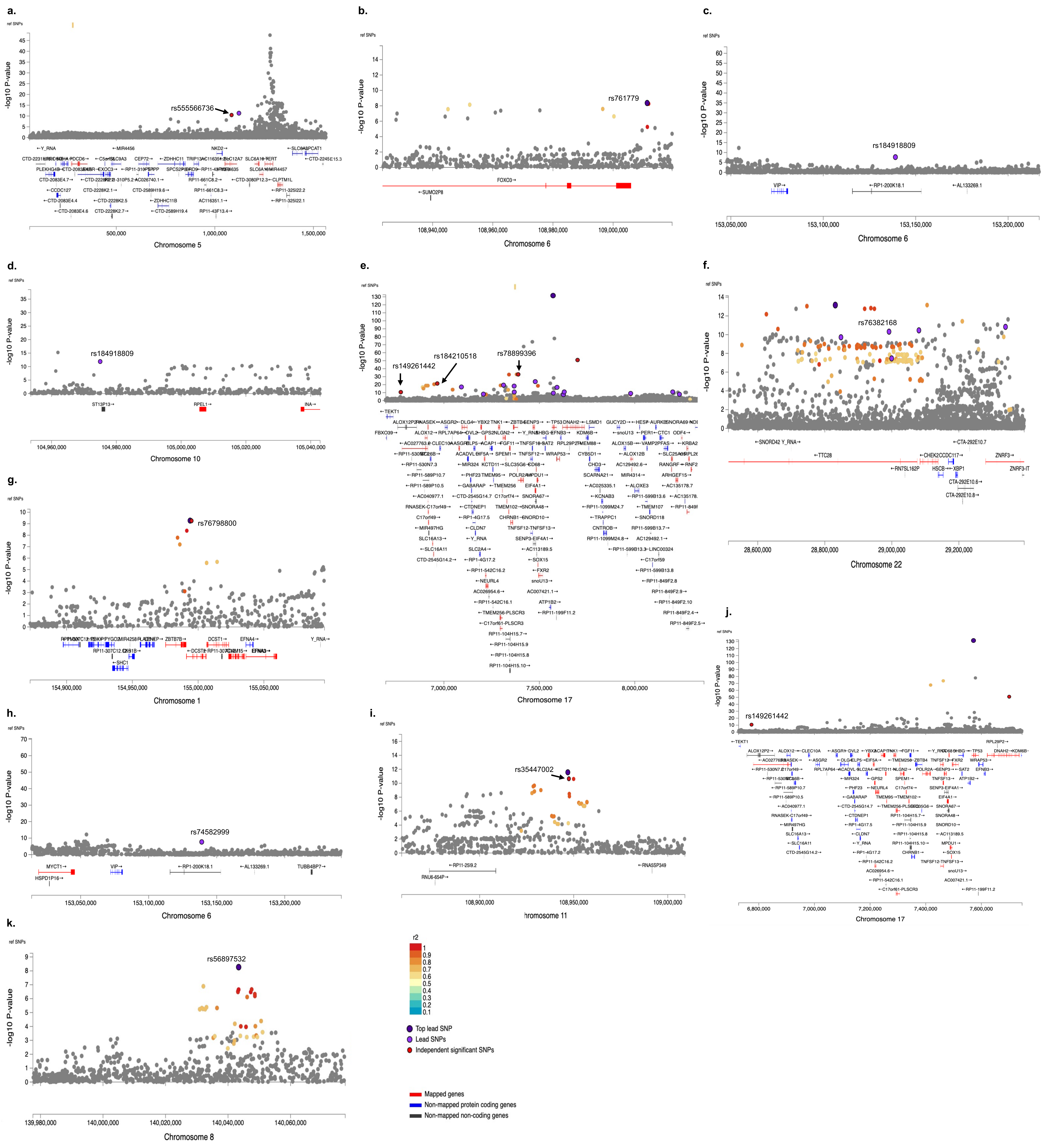

Supplementary Figure 6. Regional plots of significant novel and unpublished in previous literature locus and sentinel SNPs. a-f = Multi ancestry results. g-j = European analysis results. k = African ancestry results. Logistic regression statistical tests; multiple testing correction p-value threshold used ($5x10^{-8}$).
