# Supplementary material for: Genome-wide meta-analysis identifies novel risk loci for uterine fibroids within and across multiple ancestry groups
Source: Nat Commun. 2025 Mar 6;16:2273. doi: 10.1038/s41467-025-57483-5 (PMC11885530; doi:10.1038/s41467-025-57483-5)
Supplement: Supplementary file 3 — Description of Additional Supplementary Files [file 41467_2025_57483_MOESM3_ESM.pdf]

Description of Additional Supplementary Files

Supplementary Data 1- Demographics table of included summary statistics and statistical tests for differences in age and BMI between cases and controls. asterisk = $p < 0.05$. NHW = Non-Hispanic White (race and ethnicity), NHB = Non-Hispanic Black (race and ethnicity), EUR = European Ancestry (genetic ancestry), AFR = African Ancestry (genetic ancestry), EAS = East Asian Ancestry (genetic ancestry), CSA = Central South Asian Ancestry (genetic ancestry). All data sources meta-analyzed together for multi-ancestry analysis. Two-sample t-test; p-value threshold (0.05).

Supplementary Data 2- Significant independent SNPs, lead SNPs and conditionally independent of lead SNPs associated with uterine fibroids. a) Multi-ancestry results, b) European ancestry results, c) EastAsian/Central South Asian results, d) African ancestry results. Logistic regression statistical tests; multiple testing correction p-value threshold used (5x10-8).

Supplementary Data 3 -Significant independent and novel SNPs associated with uterine fibroids. a) Multi-ancestry results. b) European ancestry results, c) East Asian/Central South Asian results, d) African ancestry results. Logistic regression statistical tests; multiple testing correction p-value threshold used (5x10-8).

Supplementary Data 4- Genomic inflation factors, linkage disequilibrium score regression (LDSC) intercepts, and SNPbased heritability estimates of meta-analyses. LDSC statistical test for genomic inflation and LDSC intercepts, SumHer Linkage Disequilibrium Adjusted Kinships (LDAK) for LDAK heritability.

Supplementary Data 5 -FUMA Significant Gene Ontology Biological Pathway enrichment of significant variants. a) Multiancestry results. b) European ancestry results, c) East Asian/Central South Asian results. Hypergeometric statistical tests; adjusted p-value with threshold (0.05).

Supplementary Data 6 -FUMA Significant GWAS catalog enrichment of significant variants. a) Multi-ancestry results. b) European ancestry results, c) East Asian/Central South Asian results. Hypergeometric statistical tests; adjusted p-value with threshold (0.05).

Supplementary Data 7- Combined GPGE results with p-value 0.8 used for colocalization.

Supplementary Data 8- IPA results of significant network enrichment. Hypergeometric distribution testing with righttailed Fisher's Exact Test; p-value threshold used (0.05).

Supplementary Data 9- IPA analysis of significant pathway enrichment. Hypergeometric distribution testing with righttailed Fisher's Exact Test; p-value threshold used (0.05).

Supplementary Data 10 -IPA analysis of upstream regulators of data provided. Hypergeometric distribution testing with right-tailed Fisher's Exact Test; p-value threshold used (0.05).
