# Supplementary material for: Genome-wide meta-analysis identifies novel risk loci for uterine fibroids within and across multiple ancestry groups
Source: Nat Commun. 2025 Mar 6;16:2273. doi: 10.1038/s41467-025-57483-5 (PMC11885530; doi:10.1038/s41467-025-57483-5)
Supplement: Supplementary file 14 — Reporting Summary [file 41467_2025_57483_MOESM14_ESM.pdf]

nature portfolio

Corresponding author(s): Jacklyn N Hellwege

Last updated by author(s): 1/9/2025

Reporting Summary

Nature Portfolio wishes to improve the reproducibility of the work that we publish. This form provides structure for consistency and transparency in reporting. For further information on Nature Portfolio policies, see our Editorial Policies and the Editorial Policy Checklist.

Statistics

For all statistical analyses, confirm that the following items are present in the figure legend, table legend, main text, or Methods section.

n/a	Confirmed	
☐	☒	The exact sample size (*n*) for each experimental group/condition, given as a discrete number and unit of measurement
☒	☐	A statement on whether measurements were taken from distinct samples or whether the same sample was measured repeatedly
☐	☒	The statistical test(s) used AND whether they are one- or two-sided<br>*Only common tests should be described solely by name; describe more complex techniques in the Methods section.*
☐	☒	A description of all covariates tested
☐	☒	A description of any assumptions or corrections, such as tests of normality and adjustment for multiple comparisons
☐	☒	A full description of the statistical parameters including central tendency (e.g. means) or other basic estimates (e.g. regression coefficient) AND variation (e.g. standard deviation) or associated estimates of uncertainty (e.g. confidence intervals)
☐	☒	For null hypothesis testing, the test statistic (e.g. *F*, *t*, *r*) with confidence intervals, effect sizes, degrees of freedom and *P* value noted<br>*Give P values as exact values whenever suitable.*
☐	☒	For Bayesian analysis, information on the choice of priors and Markov chain Monte Carlo settings
☒	☐	For hierarchical and complex designs, identification of the appropriate level for tests and full reporting of outcomes
☒	☐	Estimates of effect sizes (e.g. Cohen's *d*, Pearson's *r*), indicating how they were calculated

Our web collection on statistics for biologists contains articles on many of the points above.

Software and code

Policy information about availability of computer code

Data collection	NA, all data were already collected and de-identified and provided for researchers to use.
Data analysis	All data analysis methods utilize publicly available and published software packages. The SNP-based heritability was estimated using SumHer (ldak5.2.linux). The lambda intercept was estimated using LD Score Regression v1.0.1. Conditional analyses were conducted using Genome-wide Complex Trait Analysis v1.93.0. Functional annotations of enrichment tests of GWAS results were completed using Functional Mapping and Annotation Web tool (accessed August 2023). Predicted gene expression as completed using S-PrediXcan/MetaXcan v0.7.1. Gene expression colocalization was conducted using coloc R library v5.2.2. OpenTargets Genetics was used to map variants to genes. Pathway analysis of colocalized genes was completed with Ingenuity Pathway Analysis (accessed March 2024).

For manuscripts utilizing custom algorithms or software that are central to the research but not yet described in published literature, software must be made available to editors and reviewers. We strongly encourage code deposition in a community repository (e.g. GitHub). See the Nature Portfolio guidelines for submitting code & software for further information.

Data

Policy information about availability of data

All manuscripts must include a data availability statement. This statement should provide the following information, where applicable:

- Accession codes, unique identifiers, or web links for publicly available datasets
- A description of any restrictions on data availability
- For clinical datasets or third party data, please ensure that the statement adheres to our policy

Genetic association summary statistics for each meta-analysis have been deposited in the GWAS Catalog under accession codes GCST90461957 [https://www.ebi.ac.uk/gwas/studies/GCST90461957] (multi-ancestry), GCST90461958 [https://www.ebi.ac.uk/gwas/studies/GCST90461958] (European ancestry), GCST90461959 [https://www.ebi.ac.uk/gwas/studies/GCST90461959] (East Asian/Central South Asian ancestry), GCST90461960 [https://www.ebi.ac.uk/gwas/studies/GCST90461960] (African ancestry). Study-specific summary statistics for BioBank Japan [https://www.pheweb.jp/pheno/UF] and FinnGen [https://r7.finngen.fi/pheno/CD2_BENIGN_LEIOMYOMA_UTERI] are available at their respective web portals. UKBB data access can be requested [https://www.ukbiobank.ac.uk/enable-your-research/apply-for-access]. BioVU [https://victr.vumc.org/how-to-use-biovu/] and eMERGE [https://emerge-network.org/collaborate/] data also require approved access, which can be requested at their respective links. The All of Us [https://www.researchallofus.org/] data is accessible on the Researcher Workbench with registered access. The data from CARDIA have been deposited at dbGaP accession phs000285.v3.p2 [https://www.ncbi.nlm.nih.gov/projects/gap/cgi-bin/study.cgi?study_id=phs000285.v3.p2] and the BWHS data at dbGaP accession phs001409.v2 [https://www.ncbi.nlm.nih.gov/projects/gap/cgi-bin/study.cgi?study_id=phs001409].The predicted expression models [https://predictdb.org/post/2021/07/21/gtex-v8-models-on-eqtl-and-sqtl/] used are publicly available. The other data generated in this study are provided in the Supplementary Data files.

Research involving human participants, their data, or biological material

Policy information about studies with human participants or human data. See also policy information about sex, gender (identity/presentation), and sexual orientation and race, ethnicity and racism.

Reporting on sex and gender	The data sources used in this varied on inclusion as self-reported female sex or woman gender. Fibroids are a disease unique to individuals with a uterus.
Reporting on race, ethnicity, or other socially relevant groupings	Our data stratification included use of EHR and self-reported race and ethnicity. We recognize that race and ethnicity are not ideal substitutes for genetic ancestry. We delineate in supplemental table 1 whether each data source uses race and ethnicity or genetic ancestry to determine the meta-analysis strata grouping. Non-Hispanic Black individuals were part of the African ancestry analyses, Non-Hispanic White and Finnish individuals were part of the European ancestry analyses, and Japanese individuals were part of the East Asian/Central South Asian analyses.
Population characteristics	Case and control definitions for each data source varied, with a range of use of ICD billing codes, CPT codes, survey response, imaging-confirmed, and self-report. These are described in the methods portion of the paper. Mean age and other population characteristics are also shown in Supplemental Table 1.
Recruitment	Each data source used had its own recruitment method, which have all been previously published. There are both hospital and community recruitment methods across the data sources. There is some ascertainment bias due to these different recruitment methods, but heterogeneity measures revealed sufficiently homogeneous meta-data
Ethics oversight	This research was approved by the Vanderbilt University Medical Center Institutional Review Board.

Note that full information on the approval of the study protocol must also be provided in the manuscript.

Field-specific reporting

Please select the one below that is the best fit for your research. If you are not sure, read the appropriate sections before making your selection.

☒ Life sciences ☐ Behavioural & social sciences ☐ Ecological, evolutionary & environmental sciences

For a reference copy of the document with all sections, see nature.com/documents/nr-reporting-summary-flat.pdf

Life sciences study design

All studies must disclose on these points even when the disclosure is negative.

Sample size	We report the complete sample size for each data source and combined meta-analyses. No sample size calculation was performed due to use of all available data.
Data exclusions	Genetic quality control excluded low quality samples and was performed separately by each data source used. Samples were also excluded if consent had been revoked, sample was duplicated, or sex concordance checks failed.
Replication	Our study was able to replicate other previously reported fibroid-associated variants and genes. Each analysis was performed independently as the cohorts do not have overlapping individuals, and each meta-analysis only includes independent data sets.
Randomization	This is an observational study of associations between genetic variants and uterine fibroids, and randomization is not required as cases and

Randomization	control definitions are based on presence or absence of fibroids.
Blinding	Blinding is not relevant to genome-wide association studies because the case control statuses are already determined, and the subsequent statistical analyses performed in this study.

Reporting for specific materials, systems and methods

We require information from authors about some types of materials, experimental systems and methods used in many studies. Here, indicate whether each material, system or method listed is relevant to your study. If you are not sure if a list item applies to your research, read the appropriate section before selecting a response.

Materials & experimental systems

n/a	Involved in the study
☒	☐ Antibodies
☒	☐ Eukaryotic cell lines
☒	☐ Palaeontology and archaeology
☒	☐ Animals and other organisms
☒	☐ Clinical data
☒	☐ Dual use research of concern
☒	☐ Plants

Methods

n/a	Involved in the study
☒	☐ ChIP-seq
☒	☐ Flow cytometry
☒	☐ MRI-based neuroimaging

Plants

Seed stocks	*Report on the source of all seed stocks or other plant material used. If applicable, state the seed stock centre and catalogue number. If plant specimens were collected from the field, describe the collection location, date and sampling procedures.*
Novel plant genotypes	*Describe the methods by which all novel plant genotypes were produced. This includes those generated by transgenic approaches, gene editing, chemical/radiation-based mutagenesis and hybridization. For transgenic lines, describe the transformation method, the number of independent lines analyzed and the generation upon which experiments were performed. For gene-edited lines, describe the editor used, the endogenous sequence targeted for editing, the targeting guide RNA sequence (if applicable) and how the editor was applied.*
Authentication	*Describe any authentication procedures for each seed stock used or novel genotype generated. Describe any experiments used to assess the effect of a mutation and, where applicable, how potential secondary effects (e.g. second site T-DNA insertions, mosiacism, off-target gene editing) were examined.*
